# Low-Cost Electronic Nose for the Determination of Urinary Infections

**DOI:** 10.3390/s24010157

**Published:** 2023-12-27

**Authors:** Alba de la Rica-Martinez, Gemma Martínez-Muñoz, Marta Amoros Sanjuan, Agustín Conesa-Celdrán, Lucía Garcia-Moreno, Gabriel Estan-Cerezo, Martin J. Oates, Nieves Gonzalo-Jimenez, Antonio Ruiz-Canales

**Affiliations:** 1 Servicio de Microbiología, Hospital General Universitario de Elche, 03202 Elche, Spain; albadelarica@gmail.com (A.d.l.R.-M.); marta.as08@gmail.com (M.A.S.); luciaizpi.garciamoreno@gmail.com (L.G.-M.); gestan@umh.es (G.E.-C.); gonzalo_nie@gva.es (N.G.-J.); 2 Engineering Department, Miguel Hernández University of Elche, 03312 Orihuela, Spainagusconesaceldran@gmail.com (A.C.-C.); moates@btinternet.com (M.J.O.)

**Keywords:** urine analysis, point-of-care, electronic nose

## Abstract

Currently, urine samples for bacterial or fungal infections require a long diagnostic period (48 h). In the present work, a point-of-care device known as an electronic nose (eNose) has been designed based on the “smell print” of infections, since each one emits various volatile organic compounds (VOC) that can be registered by the electronic systems of the device and recognized in a very short time. Urine samples were analyzed in parallel using urine culture and eNose technology. A total of 203 urine samples were analyzed, of which 106 were infected and 97 were not infected. A principal component analysis (PCA) was performed using these data. The algorithm was initially capable of correctly classifying 49% of the total samples. By using SVM-based models, it is possible to improve the accuracy of the classification up to 74% when randomly using 85% of the data for training and 15% for validation. The model is evaluated as having a correct classification rate of 74%. In conclusion, the diagnostic accuracy of the eNose in urine samples is high, promising and amenable for further improvement, and the eNose has the potential to become a feasible, reproducible, low-cost and high-precision device to be applied in clinical practice for the diagnosis of urinary tract infections.

## 1. Introduction

Urinary tract infections (UTIs) are one of the most frequent infections in the world, especially in women [1,2]. UTIs refer to the presence of microbial pathogens within the urinary tract and are usually classified by site of infection (bladder (cystitis), kidney (pyelonephritis) or urine (bacteriuria)). These infections can be asymptomatic or symptomatic, characterized by a broad spectrum of symptoms ranging from mild irritative urination to bacteremia, sepsis or even death [3].

According to the latest study on the prevalence of nosocomial infection in Spain (EPINE-EPPS 2022), in its report on the prevalence of healthcare-associated infections (HAIs), UTIs represent the third cause of infection in primary care (17.4%), after COVID-19 (23.23%) and respiratory infections (22.67%), and the second cause of HAIs (16.75%) after surgical infections (21.29%) [4]. The incidence of UTIs varies with sex and age. From 3 months of age and up to 50–65 years, women have UTIs much more frequently than men, and it is estimated that 50–60% of them will suffer from such infections throughout their lives [5]. Uncomplicated acute cystitis in young women is the most prevalent infection, with peak incidence in the years of maximum sexual activity. From the age of 65, the incidence increases in both sexes, more markedly in men, for whom it coincides with prostate disease. Moreover, 10.1% of cases of nosocomial bacteremia are secondary to UTIs, being the second focus in nosocomial bacteremia after microbiologically confirmed central vascular catheter (24.5%). In addition, 28.8% of community bacteremia cases are also related to UTIs, being the first focus in primary care patients [6].

The economic implications of UTIs are enormous because of their high incidence. Costs include doctor visits, antimicrobial prescriptions and hospitalization expenses, as well as non-medical costs associated with travel, illness and morbidity. The estimated annual cost of community-acquired UTIs in the United States is estimated to be approximately USD 1.6 billion, and the estimated annual cost of nosocomial UTIs in the United States ranges from USD 424 million to USD 451 million [3].

The etiology of UTIs depends on many factors, such as age, sex, underlying conditions, the presence of functional or anatomical disorders of the urinary tract, the community or nosocomial origin of the UTI, the use of prolonged or intermittent catheterization, history of recent hospitalization, institutionalization or previous antibiotic treatments.

*Escherichia coli* is the bacterium that is most commonly associated with UTIs. Other bacteria that can cause UTIs include *Staphylococcus saprophyticus*, *Proteus mirabilis*, *Klebsiella pneumoniae* and *Enterococcus* spp. A urine culture and an antibiogram of isolated uropathogens are essential to optimize antimicrobial treatment [6]. The required time to have a proper diagnosis can vary from 24 to 72 h between sample collection and pathogen identification.

There is a clear need for new, rapid, sensitive and reliable analytical methods, preferably with low operating costs, that can allow for the early detection of urinary tract infections and other diseases in urine. A key benefit would be the early selection of appropriate antimicrobial treatments.

Mass spectrometry and the electronic nose (eNose) are two different analytical techniques used for chemical analysis, particularly in the identification of volatile compounds or odors. They serve different purposes and have their own advantages and limitations.

Mass spectrometry is a highly versatile analytical technique used to determine the chemical composition of a sample by measuring the mass-to-charge ratio of its ions. It is capable of providing detailed information about the chemical structure of compounds, making it suitable for the identification of specific molecules and their quantification. Moreover, it is highly sensitive and can detect a wide range of compounds, including volatile and non-volatile ones. This technique is widely used in various applications, including chemistry, biology, environmental analysis and food safety.

An electronic nose, or eNose, is an instrument that mimics the human sense of smell to detect and identify odors and volatile compounds in the air. An eNose consists of an array of sensors that respond to different chemical compounds. The pattern of sensor responses is then used to identify or classify the odor or mixture of volatile compounds. eNoses are generally less specific and less precise compared to mass spectrometry and are therefore better suited for identifying broad classes of odors rather than specific molecules.

When considering the choice between mass spectrometry and eNoses for a specific application, several aspects should be taken into account. According to specificity, mass spectrometry provides high specificity and can identify individual compounds, while eNoses provide more pattern-based responses and are better suited to identifying broad categories of odors or volatiles. Concerning sensitivity, mass spectrometry is highly sensitive and can detect trace amounts of compounds, whereas eNoses may have lower sensitivity. Taking into account cost and complexity, mass spectrometry equipment is typically more expensive and requires a higher level of expertise to operate compared to eNoses.

Considering the specific requirements of the final objective, the above are the main remarkable differences between these two devices. If it is necessary to identify and quantify specific compounds with high precision, mass spectrometry is often the better choice. If a quick and relatively inexpensive method to detect or classify odors is needed, an eNose may be more appropriate.

In some cases, a combination of these techniques may be used, where an eNose is used for initial screening, and mass spectrometry is employed for more detailed analysis when specific compounds need to be identified [7].

The development of “electronic noses” (eNoses) in recent years has focused on the quality control of food products. As a result, the fragrance and food/beverage industries (in which human sensory panels are often used) have already begun to benefit from artificial nose technology. In addition, other applications have been developed for the detection of explosives [8], but the main field for these devices is the biomedical diagnosis for gastrointestinal diseases [9] or even cancer [10]. Artificial smelling may have a considerable impact on medicine in the near future; for example, research is underway in areas such as breath or urine analysis [11].

The electronic nose plays a fundamental role in relation to the characterization of samples according to their aroma, being an alternative to conventional sensory methods, as well as gas chromatography techniques. eNose technology is based on a series of non-specific sensors which react to gases and generate different signals. These signals can be processed and used for compound identification and sample classification depending on the emitted aromas. These aromas are associated with the chemical detection of complex gas mixtures, which consist mainly of volatile organic compounds (VOCs). Electronic noses are capable of detecting complex mixtures of volatile compounds present in gaseous samples. These mixtures generate a combined response in the sensors and create an odor pattern. The use of data analysis, such as principal component analysis (PCA), cluster analysis and classification techniques such as artificial neural networks (ANN) or support vector machines (SVM), have the potential to classify samples based on their aroma with a proper level of accuracy (between 70 and 100%) [12,13,14].

Currently, numerous studies have been carried out on the application of low-cost electronic noses in different samples of wine [15], olive oil [16], black tea [17] or palm tree infections [18]. Moreover, these devices are very useful at an industrial level for quality control, safety and traceability [19]. Their use in urinary samples can create a new, useful tool in UTI diagnoses. Other authors have shown the use of this technology in urine [20] and urinary tract infections [5,21,22,23]. In this study, two powerful techniques for data analysis have been employed for the analysis and classification of the dataset. These techniques are principal component analysis (PCA) and support vector machines (SVM). The selection of these methods was driven by their respective strengths and their relevance to the research objectives.

PCA is an essential dimensionality reduction technique that plays a crucial role in feature engineering. It is used to transform high-dimensional data into lower dimensional representations, preserving the most critical information while eliminating redundancy. By reducing dimensionality, PCA simplifies the dataset, making it computationally more efficient and less prone to overfitting. Additionally, PCA enhances the interpretability of the data, facilitating a more intuitive understanding of the underlying patterns.

On the other hand, SVM is a robust and versatile machine learning algorithm used for classification. The choice of SVM in this study is motivated by its capacity to handle both linear and non-linear datasets effectively. SVM constructs a decision boundary that maximizes the margin between data points of different classes, making it a powerful tool for separating complex and overlapping data. SVM can handle small datasets with high dimensionality effectively. It is particularly useful when the number of features is comparable to or even exceeds the number of data points [24]. Furthermore, SVM exhibits resilience against overfitting, which is vital for producing reliable and generalizable models. Its ability to handle high-dimensional data and non-linear relationships makes SVM an excellent choice for the experiment, where the underlying data structure may be complex and multi-dimensional.

The objective of this work is focused on adjusting, optimizing and carrying out an initial validation of a prototype of an electronic nose (eNose) device as a point-of-care diagnostic method through systematic laboratory studies and then comparing it with the current gold standard method for the diagnosis of urine infections (urine culture). To achieve this aim, the following specific objectives have been developed:

- To quantify the operational limits for the eNose as a device for the detection of bacterial or fungal urine infections;
- To validate the effectiveness and precision of the eNose in a clinical environment for the point-of-care diagnoses of bacterial or fungal infections in urine.

## 2. Materials and Methods

### 2.1. Sample Selection

During the study, 203 urine samples were processed, the selection of which was made via consecutive sampling of urine samples following requests for urine culture received at the Microbiology Service of the Hospital General Universitario of Elche (HGUE), Elche, Spain. The hospital provides coverage for the health department that encompasses the municipalities of Elche and Santa Pola, with a total population of 163,576 inhabitants. Samples from patients derived from primary care, emergency and inpatient care were included. No age restrictions were applied; the average age was 61 years (age range 4–96 years), and 62.62% of patients were female.

Urine culture requests that met the collection criteria established by the Spanish Society of Infectious Diseases and Clinical Microbiology (SEIMC) were analyzed. Sterile 100 mL bottles or 10 mL vials with or without boric acid were used. Samples with less than 5 mL of urine were excluded. Urine samples were analyzed in parallel using urine culture and through eNose technology.

#### Urine Culture Analysis

Urine culture was performed quantitatively using 1 µL calibrated loops. Each isolated colony corresponded to 1000 CFU/mL. The incubation was carried out at 35–37 °C in an enriched CO_2_ atmosphere in a blood agar base and at the same temperature under an aerobiosis atmosphere in cystine–lactose–electrolyte-deficient agar (CLED agar). The interpretation of the urine culture results was carried out according to the original criteria proposed by Kass, considering as urinary infections those that presented ≥ 100,000 colony-forming units (CFU) in the culture.

### 2.2. eNose Components and Performed Analysis

**Figure 1:**
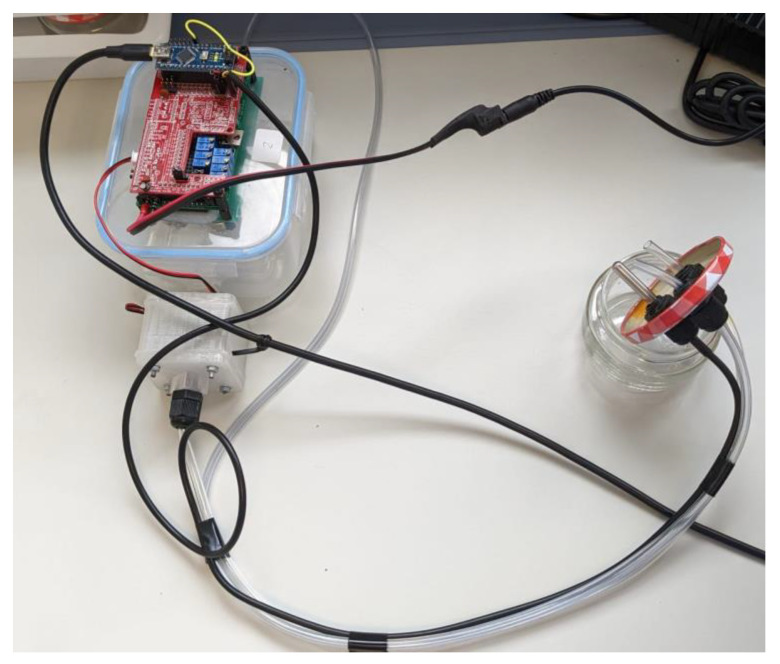
Image of the employed eNose device.

The same urine samples were analyzed using a prototype of a low-cost electronic nose (eNose), which was designed by the work group of the Engineering Department of the Miguel Hernandez University of Elche (UMH) in collaboration with the technology-based company Telenatura EBT, S.L., located in Elche (Spain). The employed device is made out of a simple sample delivery system, composed of a sample chamber, where the samples were deposited, together with an air pump or fan, as well as a matrix of sensors and a data-processing unit or microcontroller (Arduino Nano microcontroller with USB serial connection, Figure 1).

The sensor array consists of eight MQS sensors (MQ-135, MQ-2, MQ-3, MQ-4, MQ-5, MQ-7, MQ-8 and MQ-9), manufactured by Hanwei Electronics. Co., Ltd. (Zhengzhou, China), which have resistors (RLs) that change their values in function of the analyzed mixture of gases. MQSs are “Metal Oxide Semiconductor” resistive sensors. These are a type of gas sensor commonly used to detect the presence of various gases in the surrounding environment. These sensors are based on the principle that the electrical resistance of a metal oxide material changes when it comes into contact with specific gases. They are widely used in applications like air quality monitoring, industrial safety and gas leak detection.

MQ gas sensors are electrochemical sensors, and their resistance varies when exposed to certain gases. The sensors’ interior consists of a heater that is responsible for increasing the internal temperature. Thanks to the increase in heat, the sensor reacts with the gases, causing a change in the resistance value. The reason for using these sensors is their wide range of detection of VOCs (see Table 1). This prototype has been properly used for agro-food purposes in previous experiments [15,16,18].

The entire device includes several parts: eight gas sensors (MQ7 and MQ9), an Arduino Nano microcontroller and an analog circuit for controlling the heating of the sensors. The sensitivity of each sensor can be improved by modulating the voltage across the sensors and varying the input voltage sinusoidally. Then, the system modulates the heating of the sensors by varying the voltage across them. A microprocessor Arduino Nano generates voltage signals for the sensors and measures their responses. An analog circuit, including a DAC and operational amplifiers, effectively controls the sensor heating. Moreover, the voltage variation strategy includes a data sheet for some sensors. Specifically, data sheets for some sensors (MQ7 and MQ9) recommend a specific voltage switching strategy (5.0 V and 1.4 V) in a 60 + 90 s cycle, with a 90 s sensor response time.

In this work, a sinusoidal voltage variation is implemented with a period of 128 s, ranging from 1.6 to 4.8 V, and 256 steps in each period. Additionally, the sample chamber is made of glass and connected to a separate detection chamber via PVC tubing and cable glands. The detection chamber contains the sensor assembly, and a return tube completes a hermetically closed circuit. To normalize MQ sensor outputs, 50 kΩ trimmer potentiometers are used as load resistors.

Potentiometer values are adjusted until sensor channels give a voltage difference of less than 100 mV, at least half an hour after introducing the sample.

Impedance characteristics of each sensor are balanced to counteract manufacturing variability. This system is designed for precise and controlled measurements of gases or substances detected by the MQ7 and MQ9 sensors. The sinusoidal voltage variation, along with normalization techniques, aims to enhance sensitivity and account for sensor variability.

**Figure 2:**
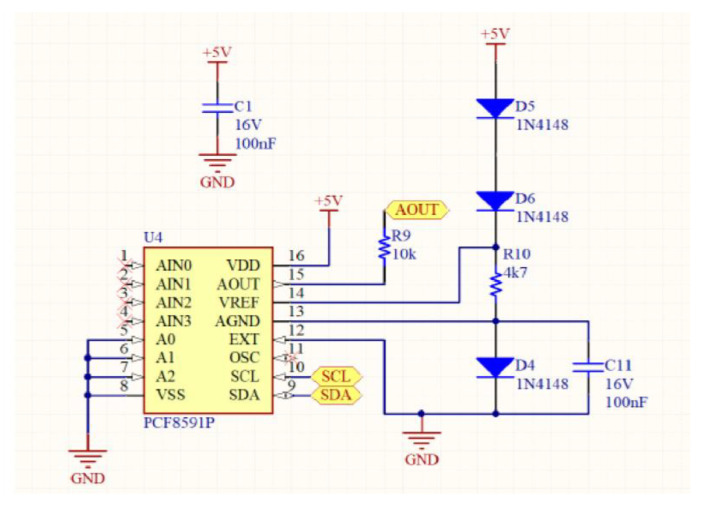
Motherboard of the device.

In addition, a second analysis is carried out to study the classification capacity of the different infections present in the group of infected samples. In Figure 2, the motherboard of the prototype device is illustrated.

On the other hand, a calibration process is needed to define the parameters of the analysis, including the stabilization time (SBT) of the sensor, prior to the start of the experiments; sensing time (ST), which represents the time that the sensors will be exposed to the sample; cleaning time (CT), which represents the time that elapses between two samples; total time (OVT = ST + CT), which represents the time necessary for the analysis of a complete sample; and the maximum number of analyses (MNA), which is estimated according to the formula MNA = (300 − SBT)/OVT. This last value is established to obtain a total time of the experiment lower than five hours, because longer times could negatively affect the results of the tests.

All experiments took place in a clean, disinfected environment. Before starting the analysis, the eNose sensors were exposed to ambient air for 30 min (SBT). After this time, a 10 mL aliquot of urine was taken, which was introduced into the sample chamber, which consisted of a 140 mL glass container. The sample chamber remained hermetically closed for 10 min (ST), during which time the sensors, contained in the lid of the container, were exposed to the sample; contact between the sensors and the liquid was avoided at all times to prevent measurement saturations. Prior to introducing the sample into the container, it was manually identified using a specific software designed by our research group.

**Figure 3:**
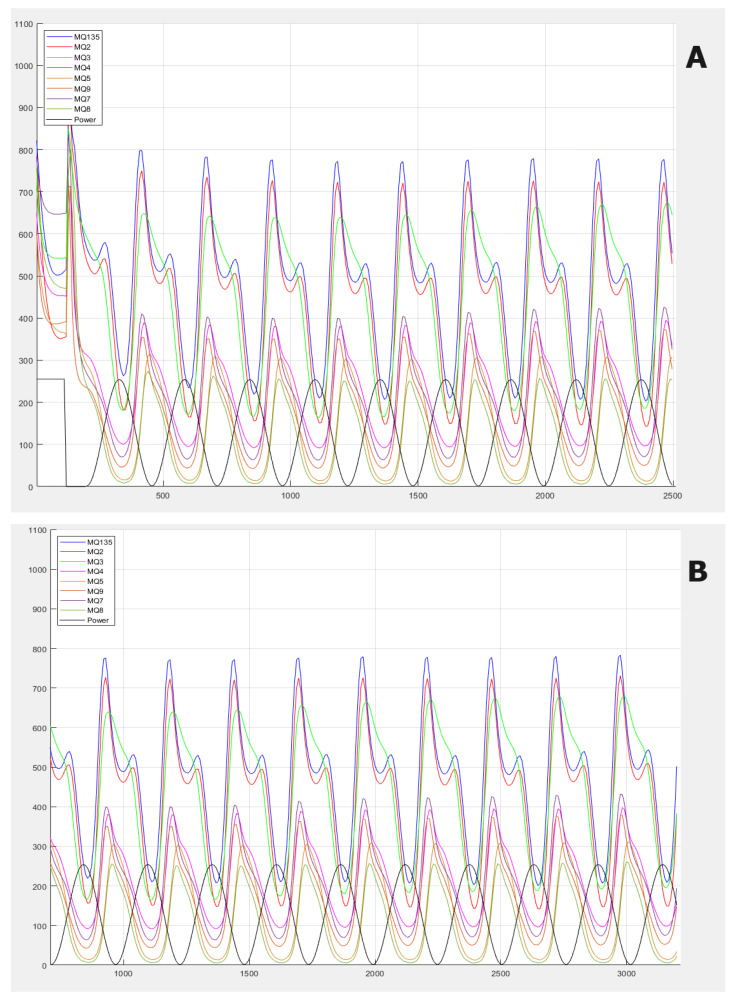

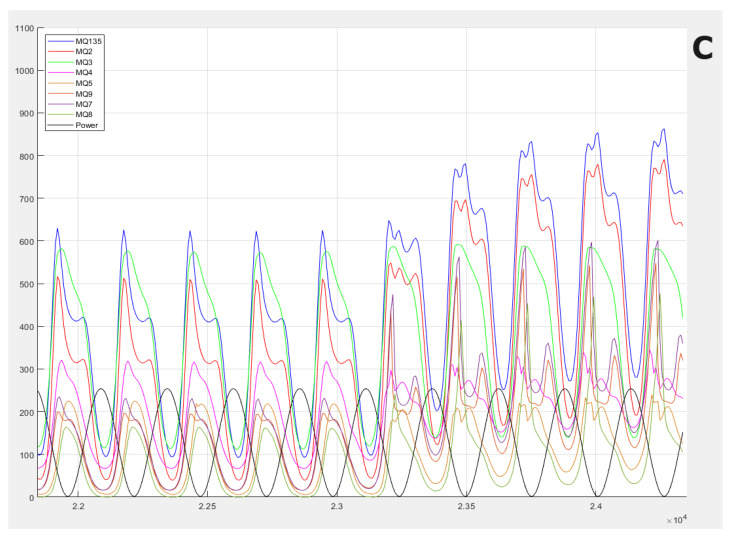
Gas information response curves of the electronic nose. (**A**) Blank sample (air). (**B**) Uninfected urine sample. (**C**) Infected urine sample (microorganism: *Proteus mirabilis*).

Once the 10 min had passed with the sample inside the jar, the sensors were again exposed to ambient air, but this time only for 20 min (CT); this time was also correctly recorded in the software developed by the research group from Miguel Hernández University of Elche (Fresh Air; version 1.0). Subsequently, successive samples were reintroduced one by one. Software was created to connect the eNose to a computer with at least 64 bits in order to obtain the data analyzed during the day, generate Excel files (.xls) containing the data of each of the daily measurements and perform an analysis of the raw data for the extraction of characteristics. The software was also capable of creating a reading of numerical data on the sample that was being analyzed at that moment, as well as the ambient air, which was stored in an Excel file automatically and simultaneously with the analysis. Once the experiment ended, the file was saved in the directory that was initially chosen, as it was a prerequisite for the analysis to choose its location and nomenclature. In Figure 3, the gas information response curves of the electronic nose are presented. The main differences between the samples are observed in terms of the frequency of the waves, which is something very difficult to see visually, and this is why we use frequency analysis techniques.

### 2.3. Data Analysis

Feature extraction was first performed to analyze the raw data. Discrete Fourier transform (DFT) was used to extract frequency coefficients from each of the temporal signals of each sensor by using the same self-developed software mentioned above. In total, 80 coefficients were extracted for each sample, thus forming a characteristic vector together with a label that was later stored and used for classification.

Secondly, a principal component analysis (PCA) was used to reduce the dimensionality of the data from 80 to 2. In this way, 2D graphs could be made, where each point represents one of the analyzed samples. With this tool, we can observe the clustering of the samples. It also allows us to select a sub-set of coefficients to be used for classifying the samples. In this case, we set the conserved variability as ≥95%, so we used 40 coefficients. The PCA and its graphic representation were executed through the Python^®^ language, using the sklearn and matplotlib libraries.

Third, the k-nearest neighbors (k-nn) algorithm was used, which is an approach for data classification that estimates the probability that a data point is a member of one group or another, according to the closest data point characteristics.

Finally, we proceed to the classification of the characteristic vectors extracted and processed via PCA by using models based on support vector machines (SVMs) in order to obtain a fast data classification tool. The reason SVM was used is that the pipeline of use of the eNose shown in this work is already established and implemented in previous works that we have developed [25]. In these, the best results obtained for the eNose data that we are dealing with have been using SVM. Also, SVM is very well studied and one of the most used algorithms for classification. It has been widely employed for eNose specific data [26,27] and thus is conclusively applicable to our purpose. Since the goal of the work is to demonstrate the applicability of the eNose prototype and the pipeline in the medical field of urinary infections, no comparison with other models is reported. The SVM model used is from sklearn library SVC C-Support Vector Classification. Parameters C and gamma for the model are obtained as explained in the text, and the kernel function is the radial basis function (RBF). Again, the kernel function integrated into the pipeline was applied. A search for the optimal values for C and gamma was performed to generate and train the models. Through a recursive code using 5-fold testing and 20% test values, values for C and gamma were obtained with a score of 0.6. The data were divided into training and testing groups, with proportions of 85% and 15%, respectively. For the binary classification between infected and non-infected samples, no specific classifier was selected. An *onevsone* (ovo) multi-classifier was used for classification among the four types of infection.

For the analysis of the results, the following confusion matrices were made for each of the models through which the total precision of the models, their sensitivity and specificity were calculated:

Accuracy = the number of hits/total number of predictions;
Sensitivity = the number of positive hits/(number of positive predictions);
Specificity = the number of negative hits/(number of negative predictions).

Combining the binary classification between infected and non-infected samples and the classification by infection, a joint model was obtained for the automatic detection of the urine infection and type of infection.

This study was approved by the ethics committee of the University General Hospital of Elche with the code PI 108/2021.

## 3. Results

### 3.1. Differentiation of Infected and Uninfected Urine

The starting point was 203 total samples, of which 108 were found to be infected (51.5% of the total) and 95 to be non-infected via urine culture. Moreover, 21 different infections were identified, of which the most common were *E. coli* (23 samples)*, Klebsiella pneumonia* (18 samples)*, Enterococcus faecalis* (18 samples) and *Proteus mirabilis* (10 samples).

**Figure 4:**
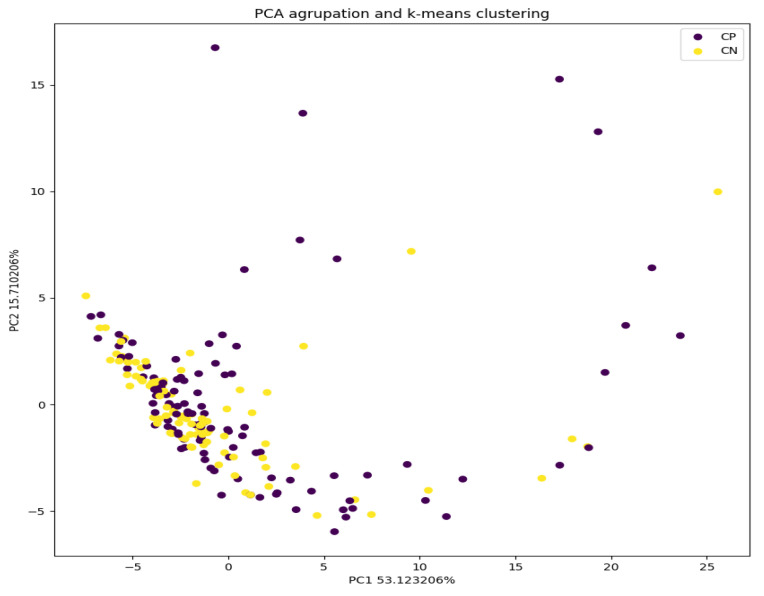
Representation of the main coefficients (PCs): control group (yellow) and infected (purple).

Figure 4 shows a graph where the first two main coefficients (PCs) that carry 69% of the variability in the data are represented. There is no clear visual differentiation between the control group (yellow) and the infected one (purple). The PC-based k-means algorithm is able to discern both groups with 51.24% accuracy and classifies the entire set, which has been entered in an unreinforced way; that is, it is capable of classifying 104 of the 203 samples correctly. In addition, visually, we can observe that within the groups, the infected group shows a greater variability between samples than the control group.

**Figure 5:**
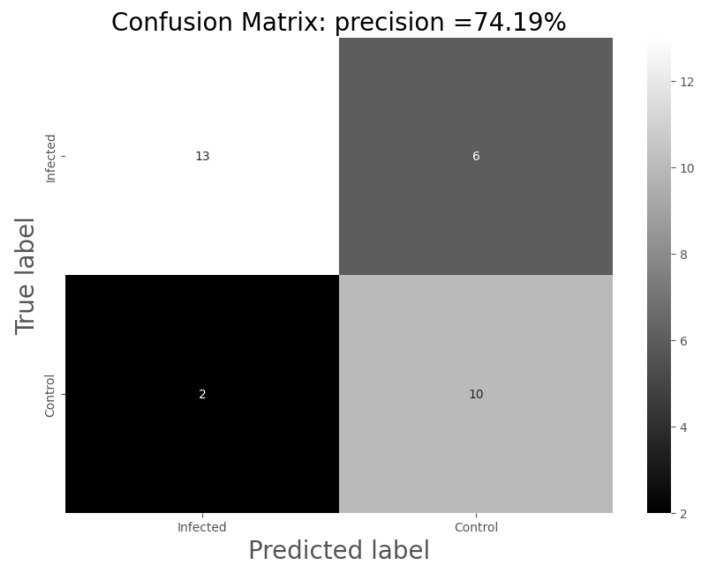
Confusion matrix after the application of SVM-based models for the control and infected variables.

It is possible to improve the accuracy of the classification, with an increase in accuracy of up to 74.19%, using SVM-based models as compared to PCA. Those models have been trained by randomly using 85% of the data for training and 15% for validation. Thus, the model was evaluated using 31 samples, of which 23 were properly classified, as the confusion matrix shows in Figure 5. The higher the number of evaluated samples is, the more reliable the obtained results will be. A higher precision when classifying non-infected samples is observed: 82% of the non-infected samples were classified correctly compared to 72% of the infected ones. This may be due to the biased training of the models, but there may also be an underlying medical relationship.

In the graph illustrating the PCA analysis in Figure 4, it can be seen that the infected samples are grouped more compactly than the uninfected ones. This is because these data are more similar to each other; that is, they present less variability. On the other hand, it is observed that the infected samples are much more dispersed in the graph, which means that they present much greater variability in the measurements. This could affect the model, increasing its capability of classifying the infected ones by making them more linked to each other as they belong to the same group.

### 3.2. Sub-Analysis by Group of Etiological Agents

**Figure 6:**
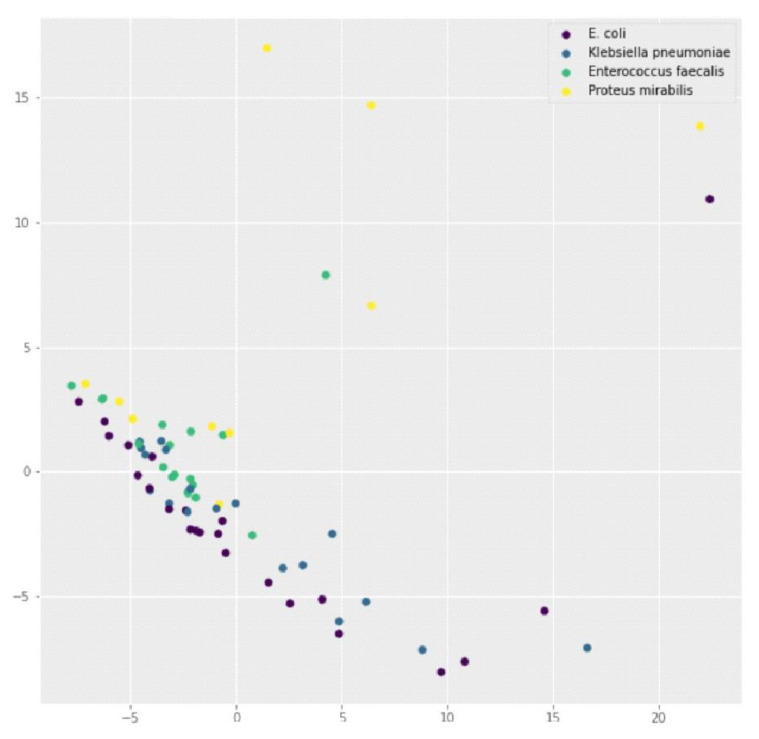
Representation of the main coefficients (PCs) of the most representative UTI-producing agents.

Figure 6 displays a graph that depicts the results of the PCA analysis on various samples of infections. The graph uses different colors to distinguish between four types of samples, as described in the figure legend. For *E. coli* (in the purple color), a diagonal aggrupation distinguished from the rest can be spotted on the bottom left of the figure. No clear differentiation can be seen between *Klebisiella pneumoniae* (in the dark blue color) and *Enterococus faecalis* (in the light blue color), which together form a diagonal clustering above the previous one. Finally, when studying the distribution of the *Proteus mirabilis* samples (in the yellow color), it is seen that they occupy a larger area of the graph and stand apart from the other samples.

**Figure 7:**
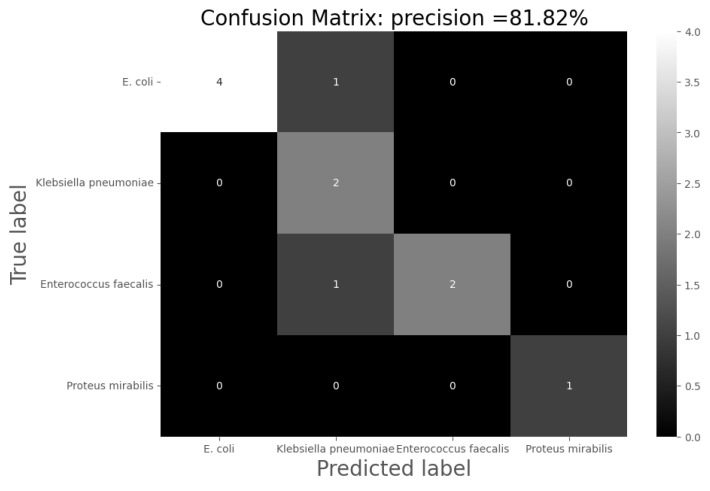
Confusion matrix after applying SVM-based models for the most representative UTI-producing agents.

The k-nn algorithm is capable of grouping the samples into the four groups of interest with 65.21% accuracy, thus classifying 45 of the 69 samples correctly according to their infection. It is possible to improve the accuracy of the classification by using SVM-based models, increasing it up 81%. To achieve this improvement, the models were trained by randomly using 85% of the data for training and 15% for validation. That is, the model was evaluated with eleven samples, of which nine were classified correctly. This can be seen in the confusion matrix shown in Figure 7. Labels 0–3 correspond to *E. coli*, *Klebsiella pneumoniae*, *Enterococcus faecalis* and *Proteus mirabilis*.

## 4. Discussion

These results demonstrate the potential of the eNose in the detection of alterations in urinary volatilome associated with urinary infection. By using models based on SVM, it is possible to improve the accuracy of the classification, increasing it up to 74%, causing a significant impact on the classification of the samples. In the present work, the accuracy of the model increased from 51.24 to 74.00% (using eNose data).

Concerning the specific prototype device used in this paper, several improvements can be applied. Mainly, it is possible to optimize the accuracy by selecting only the sensors that are involved in the detection of urine infection. This aspect was not developed initially because this device was not used previously for this purpose. Following the analysis of the sensors involved in the detection of urine infection, it will be possible to select the best sensors and add new specific sensors. This can improve the accuracy of the device and can be adapted for a commercial eNose.

eNose technology offers several significant advantages in the analysis of urinary tract infections compared to other methods. The eNose is a rapid and straightforward tool that allows for quick analysis, which can be particularly beneficial in emergency situations. In the analysis of urinary tract infections, accuracy has traditionally relied on techniques such as microbiological culture (sensitivity is 95%) [28], for which the main limitation is the time that it takes to obtain a result (24–48 h). Other methods like urinary sediment analysis (sensitivity is 90–99%) [29] require the interpretation of values and technical equipment that is not feasible to have in an emergency department. Compared to these conventional methods, the eNose technique aims to provide easily interpretable results with a point-of-care medical device suitable for integration into emergency services, thereby reducing the required time for a diagnosis. A positive result leads to an initial treatment against the infection, reducing it within 48 h, versus the usual diagnostic methods. A negative result should be validated by sending a urine sample to carry out the culturing of the sample in order to confirm the result obtained by the eNose. At least 74% of urinary tract infections will begin the treatment within 48 h. At that point, culture tests will not be substituted by an eNose, but they can be complemented. Another significant advantage of the eNose is its capability to detect a wide range of volatile compounds present in urine samples. This offers extensive coverage and enables the identification of potential infections caused by multiple pathogens. Although an accuracy rate of 74.19% is lower than in other techniques, we consider it high given the low economic cost and speed at which results are generated. Furthermore, as eNose technology continues to be developed and refined, its accuracy is likely to improve. Taking into account the aforementioned advantages and the limitations of other available methods, we believe that eNose technology holds great potential and can be a valuable tool in the analysis of urinary tract infections. However, as always, further studies and validations are necessary to support and further enhance the results obtained thus far.

In addition, it should also be noted that the model classification rate is highly dependent on the sample size. For this reason, we consider it necessary to carry out more studies to continue training the algorithm and achieve greater sensitivity and specificity.

In the graph produced by the PCA analysis in Figure 4, it can be seen that the infected samples cluster more compactly than the uninfected ones. This can be understood as these data being more similar to each other; that is, they present less variability. On the other hand, it is observed that the uninfected samples are much more dispersed in the graph, which means that they present much greater variability in their measurements. This could affect the model, making it more capable of classifying infections by being able to link them better as part of the same group.

The percentage of non-infected samples that were properly classified was higher (82% instead of 72% of infected ones). This can be explained by the basis of eNose recognition because it is based on a complex interpretation of a multi-variate non-selective response to VOCs. There are various reasons for the limitations found in the present study. A possible explanation could be urinary infections produced by microorganisms not detectable by traditional cultures, such as *Mycobacterium tuberculosis*, *Chlamydia trachomatis* or the genus *Mycoplasma* spp. In addition, urine metabolome is highly dependent on diet, environment and lifestyle, and consequently exhibits greater daily variability than serum or plasma [30]. It is also known that many types of pathologies are characterized by a certain odor and consequently, by a set of volatile substances. Many substances that are metabolites in pathogenic processes are found in human secretions, and some authors state that ethanol, butanol and their oxidation products (acetic and butyric acids) are present in almost all samples [31]. Those authors, who also measured VOCs in urine with a different device, when analyzing their results to predict the bacterial contamination of urine samples, observed that the group of samples without bacteria in the urine was more dispersed, which is similar to the observations made in our study. The authors associate this dispersion with additional factors, such as the presence of mucus and salts in the urine, which affects the redistribution of volatile substances at the “gas-liquid” phase boundary and thus the results obtained through an analysis using a series of sensors. It is very likely that further research on urine characterization will improve the correct classification of infected/uninfected samples by removing these confounding factors.

Nowadays, in clinical practice, it is quite common to prescribe antibiotics, even when there is no clear indication that it is a bacterial etiology. This is because there is no diagnostic tool that can quickly determine the presence of microorganisms and their sensitivity to various groups of antibiotics [31]. Unnecessary consumption of antibiotics leads to an increase in antimicrobial resistance (AMR) [32], which is a major health problem recognized by leading professional bodies around the world, including the World Health Organization (WHO). The current estimated annual global deaths attributed to AMR is 700,000, and this number is projected to rise rapidly and reach a worryingly high number of 10 million deaths per year by 2050. In addition to the direct impact on human health, AMR is associated with a significant global economic burden, with rising healthcare costs related to hospitalization and drug use [33].

A targeted antibiotic treatment directly implies a decrease in resistance produced by microorganisms due to inadequate treatments, as well as better therapeutic management of the patient and also the avoidance of repeat visits to health services. Diagnostics tools that make it possible to quickly and cheaply identify urinary tract infections will be of great help in these aspects.

Diagnostics are a vital part of the healthcare infrastructure, with substantial impacts on the treatment of individual patients, as well as public health policy [34]. eNose technology meets the main requirements of this type of technique. The sensors meet a number of criteria such as broad selectivity, high sensitivity and fast response and recovery time. In addition, the device can be easily reproduced, is accessible, consumes less power and has fewer consumables [20].

Despite the fact that the results for differentiating the etiological agent producing the infection are not very robust so far, they represent a good precedent for increasing the precision of this diagnostic test. The results obtained can probably be improved by increasing the number of samples used for system training. Therefore, further research on this topic is necessary. In addition, the production of certain VOCs is characteristic of bacteria [11], so it is very likely that perfecting this technology will allow us to diagnose other infectious pathologies in the future by measuring the emissions of these compounds in different biological fluids, such as pleural fluids, bronchoalveolar lavages or joint fluids. Research in this field is necessary to find new lines of diagnosis that speed up the optimization of antimicrobial treatments.

The high prevalence of urinary tract infections makes this pathology a good objective for improving the healthcare system. The identification of new and precise diagnostic tools capable of selecting patients who need antibiotic treatment continues to be a fundamental issue in the healthcare field. The economic percentage that it represents for any health system suggests that investment in advances aimed at optimizing the therapeutic management of this disease will have a positive impact in a short time.

## 5. Conclusions

In closing, the diagnostic accuracy of eNoses for specific VOCs in urine samples is high, promising and amenable for further improvement.

The presented eNose prototype has the potential to become a feasible, reproducible, low-cost and high-precision device to be applied in clinical practice for the diagnosis of urinary tract infections.

The application of this technology at point of care can provide great benefits at the clinical level, improving the quality of care at the health level, with fewer unnecessary costs, while reducing the generation of antimicrobial resistances.

The results obtained in this study highlight the need for further research to characterize urine VOCs to improve the specificity of the technique and to obtain a sensitive and precise device that is very useful in the pathology of urinary tract infections.

Subsequent experiments have to be developed in order to quantify and validate this technology. Firstly, the distinction between non-infected and infected urine has been very accurately detected. Among the infected urine samples, however, the types of infection have not been well distinguished. This aspect has to be improved in future experiments.

Finally, the detection and quantification of the main implied metabolites (VOCs) have to be taken into account for the appropriate validation of this technology.

## Figures and Tables

**Table 1:** Used sensors in the eNose prototype and their sensibilities.

N°	Sensor	Sensible to
1	MQ2	LPG (liquefied petroleum gases, hydrogen and propane)
2	MQ3	Alcohol
3	MQ4	Methane
4	MQ5	Hydrogen and LPG
5	MQ7	Hydrogen and carbon monoxide
6	MQ8	Hydrogen
7	MQ9	Carbon monoxide and LPG
8	MQ135	NH_3_ (ammonia), NO_X_, alcohol, benzene, smoke, CO_2_, etc.

## Data Availability

Data are contained within the article.
